# Supplementary material for: Microfluidic Device for Simple Diagnosis of Plant Growth Condition by Detecting miRNAs from Filtered Plant Extracts
Source: Plant Phenomics. 2024 Apr 3;6:0162. doi: 10.34133/plantphenomics.0162 (PMC10988387; doi:10.34133/plantphenomics.0162)
Supplement: Supplementary 1 — Figs. S1 to S6 Tables S1 and S2 [file plantphenomics.0162.f1.pdf]

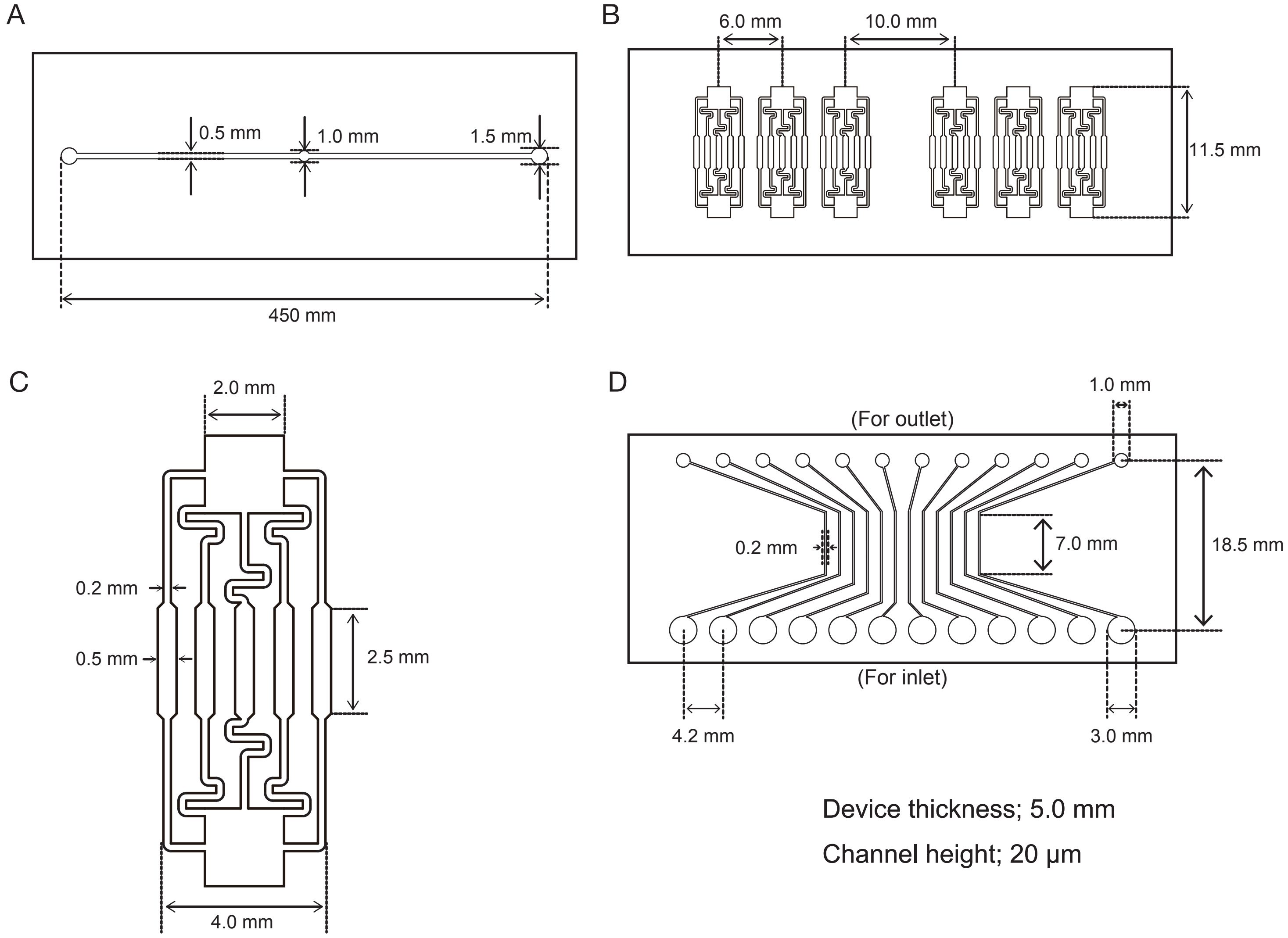

Fig. S1. Design of microfluidic devices and these channels. Dimensions of the (A) DNA probing reactor, (B) multichannel microfluidic chip, and (C) enlarged image of the detection device channels. (D) Dimension of the multichannel microfluidic chip (12 channels containing).

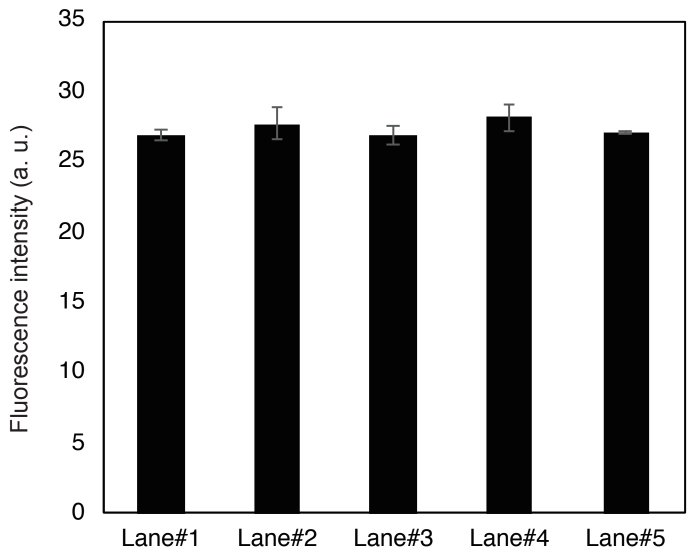

Fig. S2. Detection signals of 1 nM miR399c from the five individual channels of the detection device. This experiment was performed with a SD00011 commercial glass and NH_2- DNA probe. Error bar; SD.

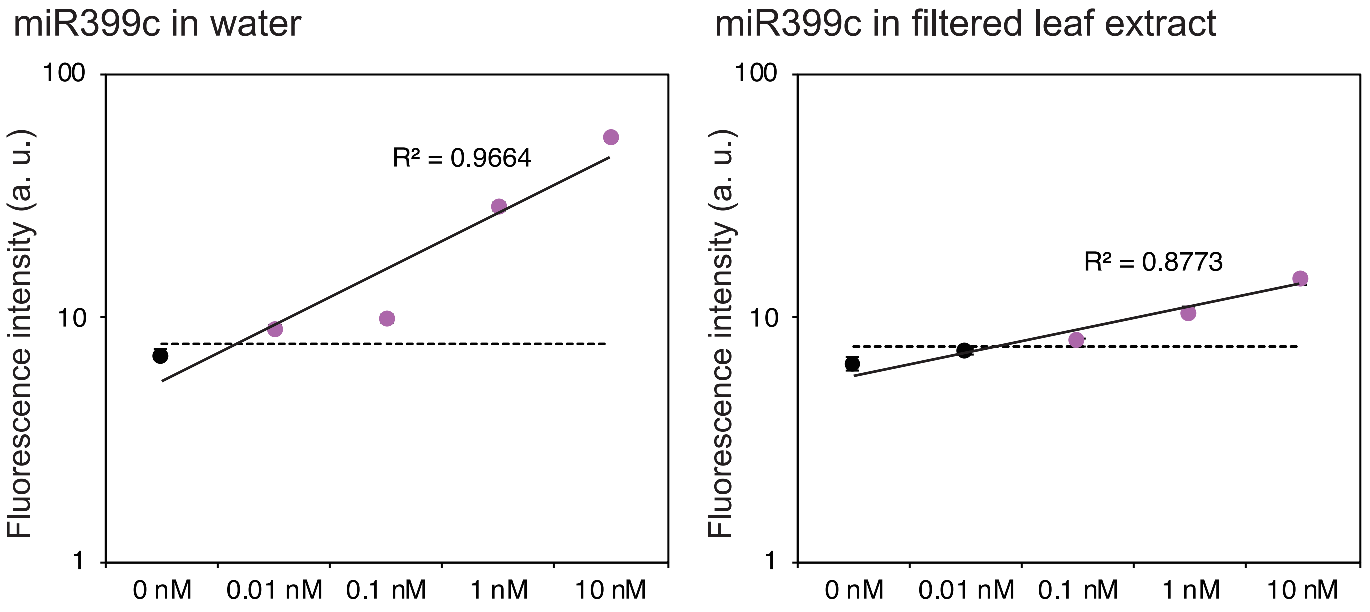

Fig. S3. Detection of different concentrations of miR399c (0-10 nM) in water or filtered extract using microfluidic device. The data were obtained from the same experiments as Fig. 4b and Fig. 5b, respectively. The dotted lines represent signal levels at three standard deviations (SDs) above the average value of 0 nM. The straight lines in the figures indicate the approximate straight lines for each data point, and their coefficients of determination (R^2) are shown above. The magenta plots indicate higher signal intensity than the dotted lines. Error bar; SD.

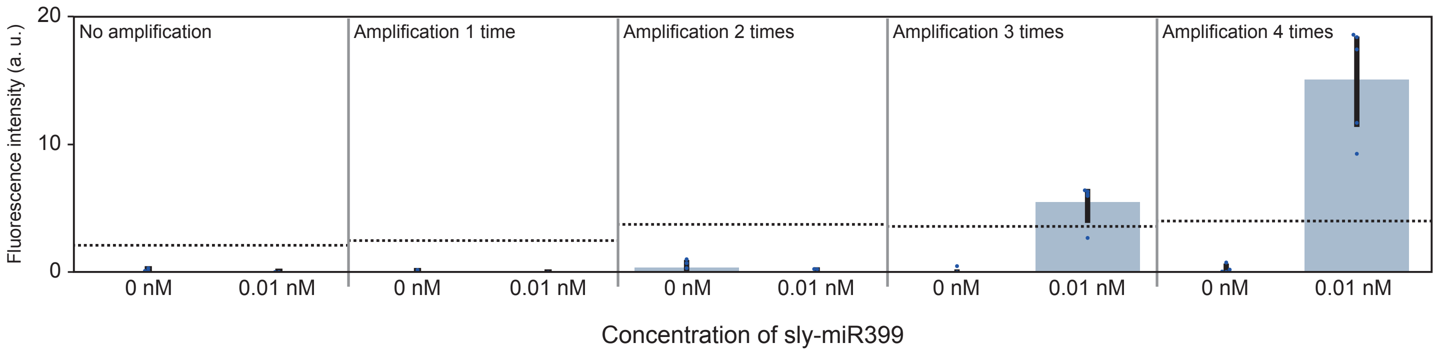

Fig. S4. Detection of artificially synthesized sly-miR399 in water by signal amplification. Detections were performed with homemade NH_2-glass and NHS-DNA probe. Blue dots and dotted lines in the graph represent each data point and the signal levels at three standard deviations (SDs) above the average of 0 nM. Error bar; SD.

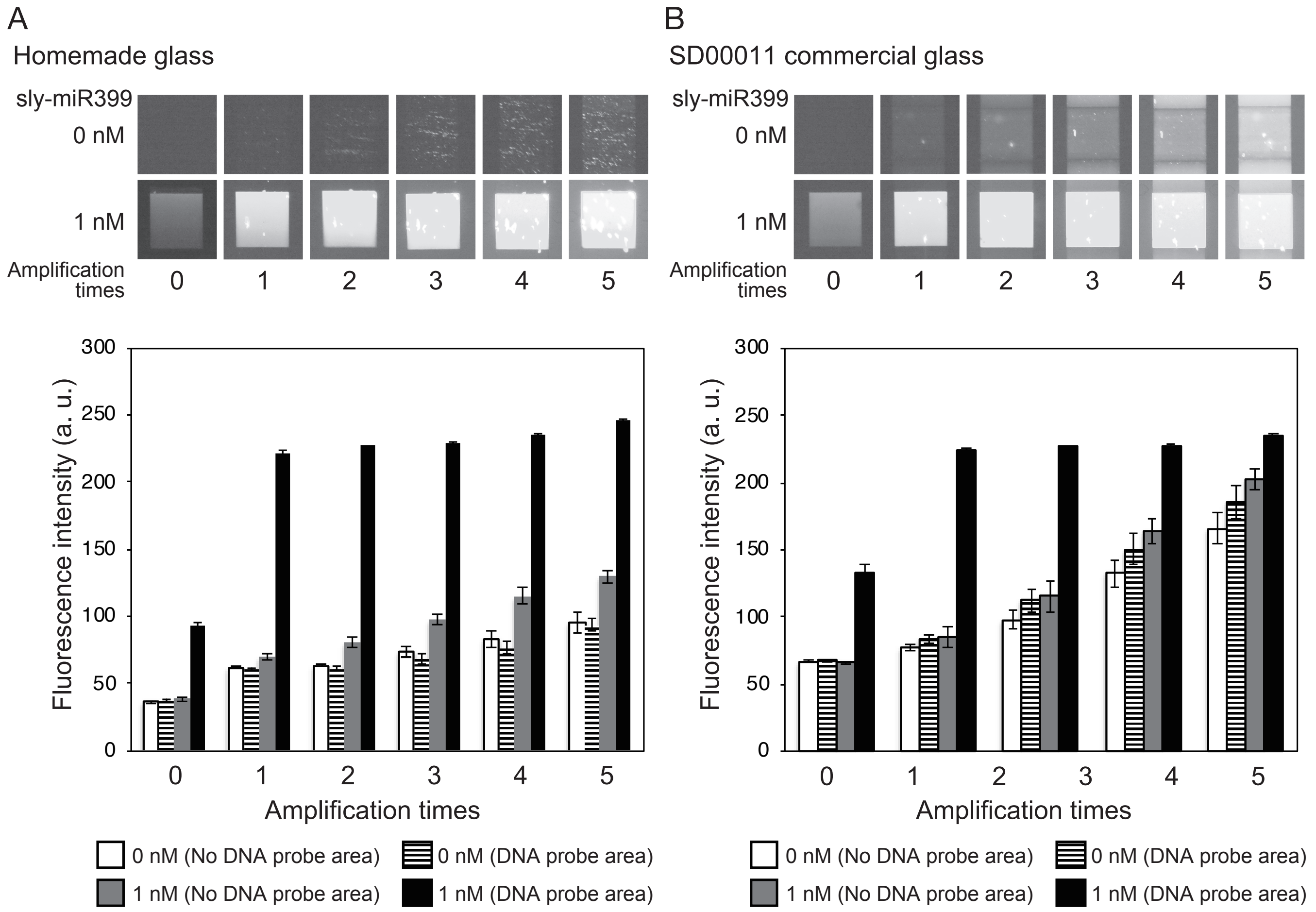

Fig. S5. Signal amplification in different amine-terminated glass. Fluorescence detection of artificially synthesized sly-miR399 by signal amplification using (A) homemade amine-terminated glass and (B) SD00011 commercial glass. The upper parts show the detection surface of the microfluidic device from no miRNA containing samples and 1 nM sly-miR399 at signal amplification by biotinylated antibody. The bar graphs show the fluorescence signals of each sample in the DNA probe or no probe area at each signal amplification times. Detections were performed with NHS-DNA probe. Error bar; SD.

A B

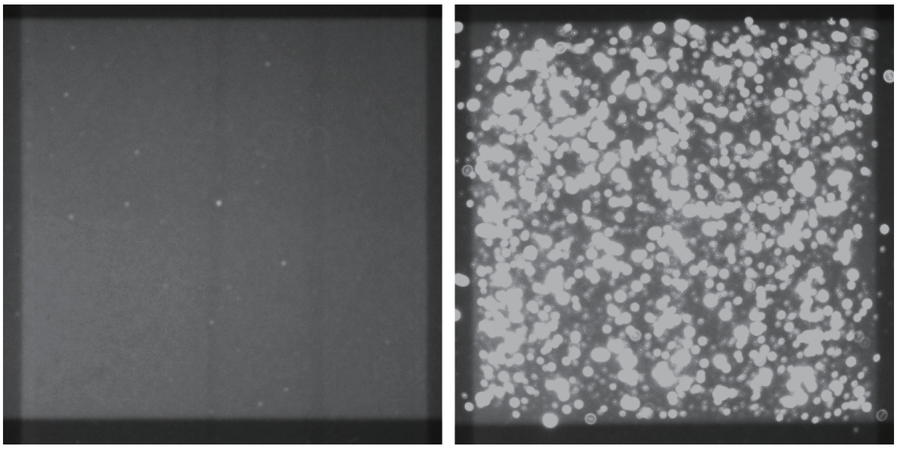

Fig. S6. Clustering of detection signals by simultaneous injection of Alexa-SA and biotinylated antibody. (A) Detection area of 1 nM miR399c with Alexa-SA only. (B) Detection area of mixed injection of Alexa-SA and biotinylated antibody after detection in (A).

Table S1. Sequences of miR399 species from Arabidopsis.

miRNAs	Sequences (5' → 3')
miR399a	UGCCAAAGGAGAUUUGCCCUG
miR399b	CCUGCCAAAGGAGAGUUGCCC
miR399d	UGCCAAAGGAGAUUUGCCCCG
miR399e	UGCCAAAGGAGAUUUGCCUCG
miR399f	UGCCAAAGGAGAUUUGCCCGG

Table S2. Sequences of primers for qRT-PCR.

	Primers	Sequence (5' → 3')
Stem loop primer	sly-miR399	GTCGTATCCAGTGCAGGGTCCGAGGTATTCGCACTGGATACGACTAGGGCAAC
Forward primers	sly-miR399_F	CGACGTTGCCAAAGGAGAGTTG
	sly-actin_F	GAGGATATTCAGCCCCTTGTTTG
Reverse primers	Universal reverse primer	CCAGTGCAGGGTCCGAGGT
	sly-actin_R	CATCTTTCTGACCCATTCCAACC
